# Health service utilization and medical costs for in-hospital death at the end of life

**DOI:** 10.1186/s12913-025-13791-6

**Published:** 2025-11-22

**Authors:** Shengnan Duan, Lina Xie, Huaxin Yu, Jiaxue Cui, He Zhao, Tong Ding, Huihua Li, Bangjun Li, Jie Yang

**Affiliations:** 1 Dongbei University of Finance & Economics, Dalian, China; 2 Dalian Medical University, Dalian, China; 3 The Center of Disease Control and Prevention, Dalian Jinpu New District (Health Supervision Institute, Dalian Jinpu New District), Dalian, China; 4 The Second Affiliated Hospital of Dalian Medical University (Puwan), Dalian, China; 5 The First Affiliated Hospital of Dalian Medical University, Dalian, China

**Keywords:** In-hospital death, Health service utilization, Medical costs, Influencing factors, End-of-life

## Abstract

**Background:**

Despite comprising < 1% of the population, end-of-life (EOL) patients consume 8%–11.2% of global health expenditures. China’s aging population and limited health service urgently require cost-factor analyses. Data from 3,323 in-hospital deaths (2021–2023) were collected to explore health service utilization and medical costs at the EOL and provide evidence-based recommendations.

**Methods:**

Based on a review of medical records from 2021 to 2023 in a tertiary hospital, data from 3,323 in-hospital deaths were compiled and analyzed. Descriptive statistics were applied to summarize demographic characteristics, health service utilization, and medical costs. Multiple linear regression analysis was performed to identify influencing factors of medical costs, including both total medical expenditures and out-of-pocket payments (OOP).

**Results:**

Patients aged ≥ 65 years accounted for 73% of in-hospital deaths and 84.5% of total medical expenditures. A positive correlation was observed between age and total medical expenditures (β = 0.002, *P* < 0.05), whereas a negative correlation was found with OOP payments (β = − 0.002, *P* < 0.05). The mean medical expenditure (64,889 yuan) was substantially higher than the 2023 public data (129,000 yuan), driven primarily by spending on pharmaceuticals (32.4%) and laboratory diagnostics (15.3%). Patients covered by urban employee insurance incurred higher total expenses, while urban-rural insurance holders experienced lower OOP burdens. Married patients exhibited greater OOP payments. Comorbidities were significant contributors to elevated total medical expenditures and OOP payments.

**Conclusions:**

China’s rapidly aging population intensifies the challenges of EOL medical and healthcare costs and service utilization. Policymakers should implement both supply- and demand-side reforms, conduct targeted educational initiatives to foster a balanced understanding of life and death, and strengthen patient-centered approaches such as long-term care insurance, home-based palliative care, and a comprehensive management system for comorbidities. In addition, measures within hospital and medical insurance reforms should be refined, supervision and regulation should be strengthened, and the participation of communities and social organizations should be actively promoted in caring for and supporting patients at the EOL. Collectively, these strategies can enhance patients’ quality of life, increase healthcare efficiency, and maintain sustainable control over medical cost growth.

## Introduction

The term end-of-life (EOL) refers to individuals clinically diagnosed as being in the terminal phase of illness, with death imminent, typically confirmed through multidisciplinary assessments involving medical and pathological evaluations [1]. Globally, the setting of death has shifted significantly; in many high-income countries, over 60% of deaths now occur in hospitals, with extreme cases reported in South Korea (84.9%) [2]. This trend has created major challenges in healthcare service utilization and increased financial strain on health systems. The Lancet reported that the treatment costs in the final months of life are a major contributor to family poverty in countries lacking universal health coverage. In several high-income countries, 8%–11.2% of total health expenditures are consumed by less than 1% of the population–those who died within that year [2]. Understanding patterns of healthcare utilization and medical costs at the EOL is increasingly vital for optimizing resource allocation and containing costs, representing a critical yet misunderstood aspect of population health.

China currently faces compounded challenges. Economic vulnerability: A study in China involving 792 cancer patients revealed that more than 80% received life-prolonging treatments. Among these patients, 84.1% of urban families and 91.1% of rural families subsequently fell into poverty [3]. Demographic pressure: The country’s large and rapidly aging population poses an increasing burden, with the proportion of elderly individuals rising from 7.0% in 2000 to 18.1% in 2023 [4]. This trend is associated with a decline in overall population health [5] and a persistently high burden of noncommunicable chronic diseases [6]. System fragmentation: Uneven regional health investments [7] and substantial disparities in per capita healthcare spending between urban and rural areas [8] continue to widen inequities, placing end-stage patients and their families at high risk of health-related poverty and financial vulnerability [9].

Consequently, investigating health service utilization and medical costs prior to in-hospital death is essential for ensuring the rational allocation of healthcare resources and the effective control of medical expenditures in China’s aging society. Such analyses can also help promote the development of hospice and palliative care at the EOL. This study analyzed data from 3,323 in-hospital deaths (2021–2023) obtained from a tertiary hospital in China with the aim of informing strategies for medical cost control and aging response under the Healthy China Initiative.

## Methods

### Study population and data sources

In-hospital death represents a key subset of the EOL population. This study included patients whose discharged outcome was death, based on inpatient records, and examined their health services utilization and medical expenditures during their final hospitalization.

Data were extracted from medical records of 3,323 deceased inpatients (2021–2023) at a tertiary hospital, including information on demographics, clinical characteristics (admission and discharge), and medical expenditures. Ethics approval for this study was obtained in accordance with the Declaration of Helsinki.

### Variables and statistical analysis

Descriptive statistics were used to describe demographics variables, health service utilization, and medical expenditures. Multiple linear regression analysis was applied to identify the determinants of medical costs (total medicalexpenditures and out-of-pocket (OOP) payments) with a significance level set at ɑ = 0.05. The variance inflation factor (VIF) was employed to test for multicollinearity among the independent variables, where a VIF value > 10 indicates the presence of multicollinearity. The dependent variables were total medical expenditures and OOP payments. Independent variables were selected based on a comprehensive literature review, expert consultations, and preliminary data exploration to ensure theoretical and empirical relevance. These included marital status, gender, occupation, ethnicity, age at death, primary causes of death, route of admission, interdepartmental transfer or not, number of surgical procedures, drug allergies, average length of hospital stay, comorbidity, and number of hospitalizations at the same facility. Disease classifications followed ICD-10-CM standards. Data were cleaned in a relational database (Microsoft Excel^®^), with validation to correct missing or inconsistent entries; records lacking a valid date of death were excluded. Statistical analyses were performed using IBM SPSS Statistics 21.0. Pearson’s χ² tests (or the Cochran-Armitage trend tests for ordinal variables) and the Mann–Whitney U test were used for non-normal data (validated by the Shapiro–Wilk test). Statistical significance was defined as two-tailed *P* < 0.05, and sensitivity analyses were conducted to ensure the robustness of results.

## Results

### Characteristics of in-hospital death

A total of 3,323 in-hospital deaths were recorded, distributed as 26.4% in 2021, 30.3% in 2022, and 43.3% in 2023. The proportion of in-hospital deaths among all hospitalized patients was 1.0% overall (0.9% in 2021, 1.0% in 2022, 1.1% in 2023). Among these, 2,151 were males (64.7%) and 1,172 were females (35.3%). Most deaths occurred among individuals aged 65 years or older, representing 73.0% of all cases, while those aged 55–64 years accounted for 14.5%. Additional details are presented in Table 1.

**Table 1:** Characteristics of In-Hospital death

Characteristics	2021(*n* = 877)		2022(*n* = 1008)		2023(*n* = 1438)		Total	Ratio(%)
	*N*	%	*N*	%	*N*	%		
**Gender**								
Male	573	65.3	654	64.9	924	64.3	2151	64.7
Female	304	34.7	354	35.1	514	35.7	1172	35.3
**Marital status**								
Single	103	11.7	117	11.6	185	12.9	405	12.2
Have partner	774	88.3	891	88.4	1253	87.1	2918	87.8
**Work or not**								
Yes	187	21.3	266	26.4	321	22.3	774	23.3
No	690	78.7	742	73.6	1117	77.7	2549	76.7
**Ethnicity**								
Han	846	96.5	958	95.0	1379	95.9	3183	95.8
Others	31	3.5	50	5.0	59	4.1	140	4.2
**Insurance**								
URRBMI	736	83.9	891	88.4	1328	92.4	2955	88.9
URRBMI Outside the city	3	0.3	0	0.0	0	0.0	3	0.1
CMI	4	0.5	2	0.2	3	0.2	9	0.3
FMS	30	3.4	7	0.7	10	0.7	47	1.4
None	100	11.4	107	10.6	95	6.6	302	9.1
Others	4	0.5	1	0.1	2	0.1	7	0.2
**Age at death**								
0 ~ 14	5	0.6	1	0.1	0	0.2	9	0.3
15 ~ 24	1	0.1	6	0.6	4	0.3	11	0.3
25 ~ 34	17	1.9	14	1.4	14	1.0	45	1.4
35 ~ 44	40	4.6	31	3.1	39	2.7	110	3.3
45 ~ 54	73	8.3	64	6.4	105	7.3	242	7.3
55 ~ 64	147	16.8	146	14.5	189	13.1	482	14.5
65 ~ 74	234	28.9	283	28.1	380	26.4	897	27.0
75 ~ 84	220	25.1	281	27.9	402	28.0	903	27.2
85~	140	16.0	183	18.2	303	21.1	626	18.8
**Primary causes of death**								
Infectious diseases and parasites	49	5.6	47	4.7	87	6.1	183	5.5
Cancer	106	12.1	88	8.7	141	9.8	335	10.1
Hematopoietic organs and immune diseases	38	4.3	32	3.2	43	3.0	113	3.4
Endocrine, nutritional and metabolic diseases	20	2.3	18	1.8	8	0.6	46	1.4
Meningitis, encephalopathy	21	2.4	19	1.9	26	1.8	66	2.0
Cardiovascular system	190	21.7	200	19.8	208	14.5	598	18.0
Cerebrovascular disease	210	23.9	198	19.6	226	15.7	634	19.1
Respiratory system	113	12.9	184	18.3	497	34.6	794	23.9
Digestive system	50	5.7	61	6.1	74	5.1	185	5.6
Musculoskeletal and connective tissue	7	0.8	8	0.8	7	0.5	22	0.7
Genitourinary system	11	1.3	32	3.2	49	3.4	92	2.8
Pregnancy, childbirth, perinatal diseases	6	0.7	1	0.1	2	0.1	9	0.3
Congenital monstrosity	2	0.2	0	0.0	1	0.1	3	0.1
Acatalepsia	10	1.1	32	3.2	18	1.3	60	1.8
Damage and poisoning	38	4.3	62	6.2	40	2.8	140	4.2
Other diseases	6	0.7	26	2.6	11	0.8	43	1.3

URRBMI: Urban and rural residents basic medical insurance

CMI: Commercial medical insurance

FMS: Free medical service

Analysis of the primary causes of death revealed that respiratory diseases (23.9%) were the most common, followed by cerebrovascular diseases (19.1%), cardiovascular diseases (18.0%), malignant tumors (10.1%), and digestive system diseases (5.6%). The distribution pattern remained relatively consistent across the three years, although with some variations. In 2021, the leading causes were cerebrovascular diseases (210, 23.9%), cardiovascular diseases (190, 21.7%), and respiratory diseases (113, 12.9%). In 2022, cardiovascular diseases (200, 19.88%), cerebrovascular diseases (198, 19.6%), and respiratory diseases (184, 18.3%) were most prevalent, whereas in 2023, respiratory diseases (497, 34.6%), cerebrovascular diseases (226, 15.7%), and cardiovascular diseases (208, 14.5%) ranked highest. In addition to the primary causes of death, 98.9% of deceased patients had secondary diagnoses, with an average of 7.1 comorbidities and a median of 8.

### Health service utilization before in-hospital death at the EOL

The primary routes of hospital admission were emergency transfers (81.1%) and outpatient transfers (18.7%). Approximately 16.4% of in-hospital deaths involved a transfer to another clinical department during their final hospitalization. Overall, 60.0% of patients underwent surgical procedures, with an average of 2.9 surgeries (median = 2). The average length of hospital stay was 10.4 days (median = 6.0) (Table 2). Among these cases, 960 in-hospital deaths (28.9%) occurred among patients from the intensive care unit (ICU), whose average length of stay was 10.2 days (median = 5.0). Annual data are summarized in Table 2.

**Table 2:** Health service utilization before In-Hospital death at the end of life

Variable	2021(*n* = 877)		2022(*n* = 1008)		2023(*n* = 1438)		Total /Mean	Median/%
	*N*/Mean	Median/%	*N*/Mean	Median/%	*N*/Mean	Median/%		
**The route of admission**								
emergency transfers	724	82.5	842	83.5	1130	78.6	2696	81.1
outpatient transfers	148	16.8	166	16.5	306	21.3	620	18.7
Others	5	0.5	0	0	2	0.1	7	0.2
**Interdepartmental transfer or not**								
Yes	133	15.2	11	1.1	236	16.4	546	16.4
No	744	84.8	997	98.9	1202	83.6	2777	83.6
**No. of surgical procedures**	1.6	1	1.9	1	1.7	1	1.8	1
**Average length of hospital stay (ALSO)**	11.4	5	8.7	5	10.9	7	10.4	6
**No. of hospitalizations at the same facility**	3.6	2	3.7	2	4.1	2	3.9	2

ALOS: Average Length of Hospital Stay

Patients aged 65 years had an average hospital stay of 10.8 days, with 82.7% admitted through emergencies and 17.3% through outpatient services. Among them, 15.7% were transferred to another clinical department, 57.6% underwent surgery, and 74.4% were discharged from the ICU.

### Medical expenditures and OOP payments before in-hospital death at the EOL

The total medical expenditure for in-hospital death was 215,625,643 yuan, accounting for 2.6% of the total medical expenditures for all hospitalized patients. The average per capita medical expenditure were 64,889 yuan. The total OOP expenses before in-hospital death at the EOL were 55,684,423 yuan, representing 25.8% of the total hospitalization costs; the average OOP payment per patient was 16,757 yuan, equivalent to 35.2% of the local per capita disposable income [10]. In 2021, the total EOL medical expenditure reached 58,868,582 yuan (2.3%) of the total hospitalization expenses, with an average of 67,124 yuan per patient. In 2022, the total was 66,297,421 yuan (2.5%), while in 2023, the total rose 65,771 yuan (2.9%) with an average medical expenditures of 62,906 yuan (Table 3). The total medical and OOP costs were 16,261,730 yuan and 4,774,557 yuan, respectively, corresponding to average values of 71,638 yuan and 21,033 yuan per patient.

**Table 3:** PCME and PC-OOP of patients with different causes of death

Variable	2021		2022		2023		Total	
	PCME	PC-OOP	PCME	PC-OOP	PCME	PC-OOP	PCME)	PC-OOP
**Age at death**								
0 ~ 14	37,290	11,029	20,153	8,116	68,913	26,306	45,927	15,798
15 ~ 24	78,854	30,380	51,090	5,468	183,574	49,754	101,790	23,837
25 ~ 34	55,967	6,913	63,790	13,814	107,190	12,151	74,337	10,690
35 ~ 44	87,817	17,086	53,752	12,436	76,296	18,182	74,133	16,164
45 ~ 54	62,435	19,992	74,804	22,841	76,536	17,981	71,824	19,873
55 ~ 64	62,279	16,488	81,137	22,697	62,715	14,316	68,162	17,516
65 ~ 74	62,877	17,085	60,081	17,220	67,445	18,447	63,970	17,718
75 ~ 84	78,149	21,132	71,852	19,231	57,041	14,922	66,793	17,776
85~	60,859	13,502	52,517	11,296	54,849	12,838	55,519	12,536
**Dignosis**								
Infectious diseases and parasites	91,314	21,669	56,894	13,640	83,840	17,003	78,920	17,389
Cancer	50,883	17,330	64,388	19,528	43,591	13,345	51,362	16,230
Hematopoietic organs and immune diseases	43,673	12,633	58,197	20,452	45,004	10,334	48,293	13,973
Endocrine, nutritional and metabolic diseases	56,300	14,775	42,120	4,716	35,206	9,071	4,7082	9,847
Meningitis, encephalopathy	133,140	22,014	38,122	6,892	40,982	7,719	69,482	12,029
Cardiovascular system	49,832	14,909	51,678	14,152	40,410	12,126	47,172	13,688
Cerebrovascular disease	54,945	16,295	76,789	25,863	74,547	21,651	68,755	21,193
Respiratory system	99,979	25,838	62,226	14,338	72,638	16,775	74,116	17,500
Digestive system	94,321	25,792	77,740	20,611	68,078	15,835	78,356	20,101
Musculoskeletal and connective tissue	50,450	14,673	74,101	21,723	77,413	22,999	67,630	19,886
Genitourinary system	75,916	19,453	36,842	10,000	30,277	6,294	38,017	9,156
Pregnancy, childbirth, perinatal diseases	35,064	9,192	20,154	8,116	88,385	34,191	45,256	14,627
Congenital monstrosity	62,682	14,712	/	/	39,036	28,864	54,800	19,429
Acatalepsia	40,743	5,984	133,849	34,152	119,732	38,569	114,096	30,783
Damage and poisoning	115,544	59,48	90,519	10,823	62,502	5,713	89,306	8,040
Other diseases	12,804	3,225	45,914	15,074	40,784	13,684	39,982	13,065

PCME: Per Capita medical costs

PC-OOP: Per Capita out-of-pocket payments

Unit: Yuan

**Fig. 1:**
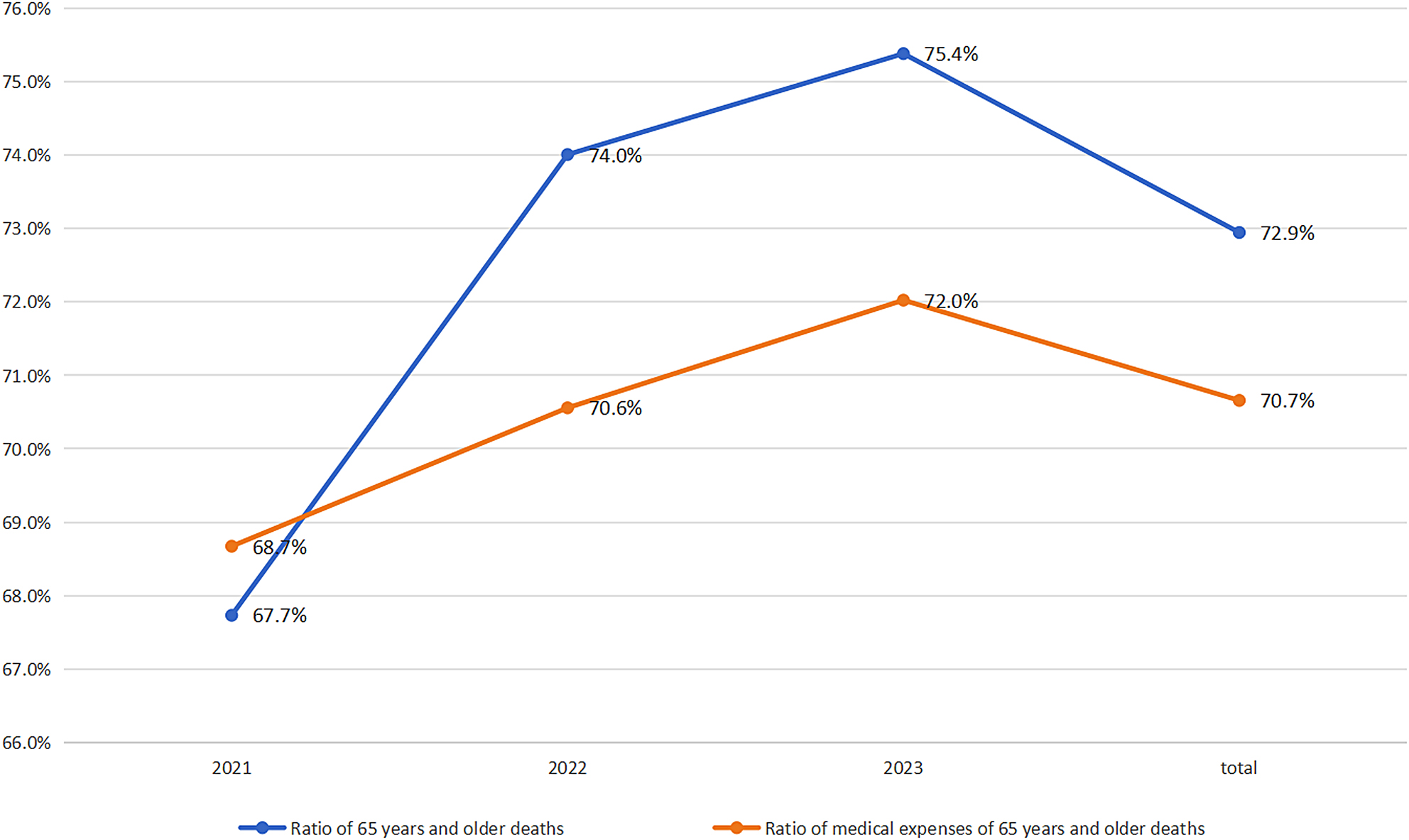
Ratio of 65 years and older deaths and their medical costs

Among patients aged ≥ 65 years, the total medical expenditure was 152,357,066 yuan (70.7%), with an average of 62,853 yuan per patient. The OOP payments for this group were 39,768,633 yuan, averaging 16,406 yuan per person. In 2021, medical expenditures for elderly decedents were 40,426,301 yuan (68.7%); in 2022, they were 46,777,797 yuan (70.6%); in 2023, they were 65,152,967 yuan (70.7%) (Fig. 1).

**Fig. 2:**
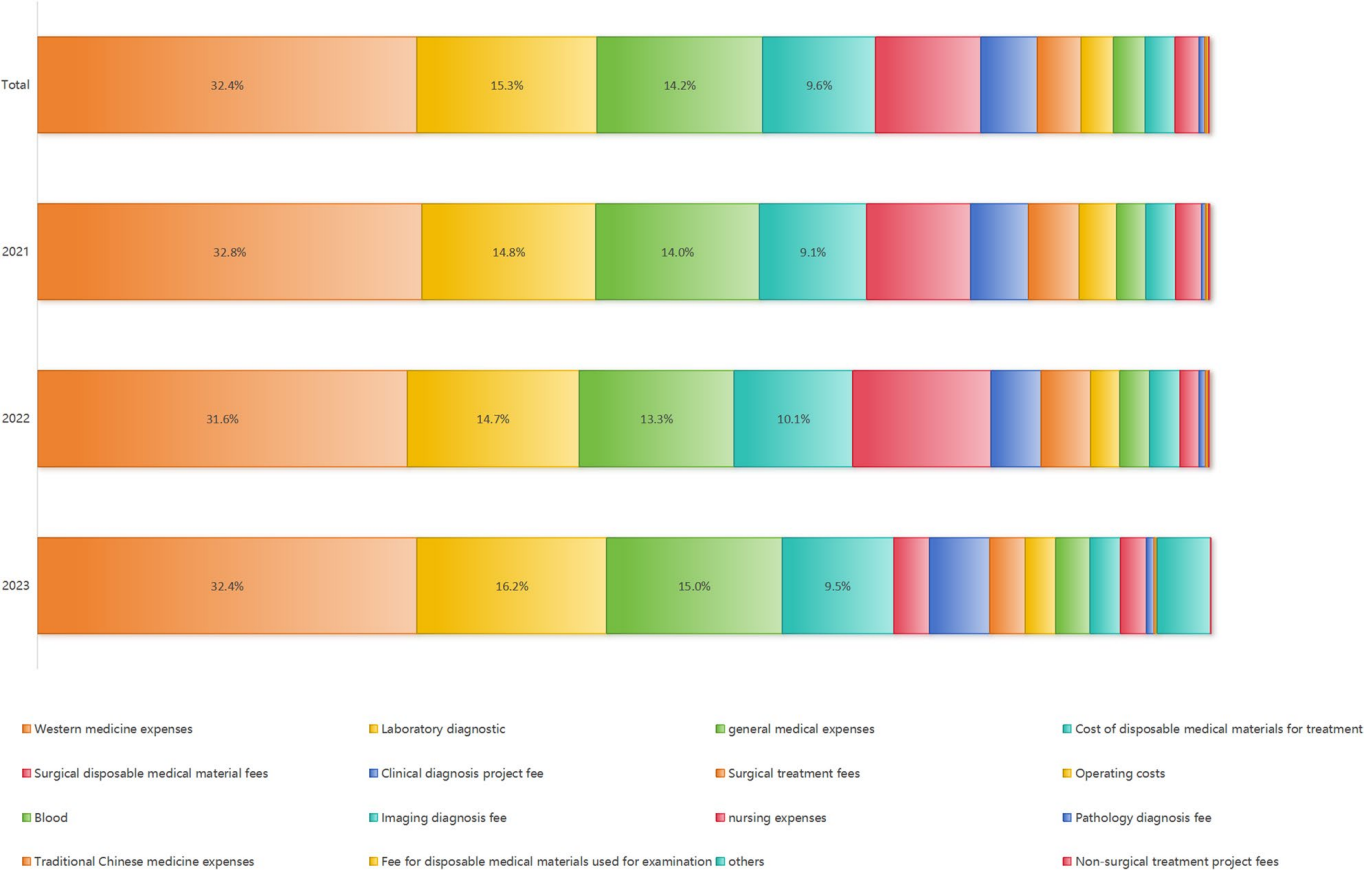
Composition of medical costs before in-hospital death

The details of medical expenditures revealed that Western medicine costs accounted for the largest share—69,890,339 yuan (32.4%), followed by laboratory diagnosis fees (15.3%), general medical service fees (14.2%), disposable medical materials for treatment (9.6%), disposable medical materials for surgery (9.0%), and clinical diagnostic project fees (4.8%). From 2021 to 2023, the proportion of expenditures on Western medicine costs, laboratory diagnostics, and general medical operation fees remained relatively stable (Fig. 2).

By disease category, excluding cases with unclear diagnoses, the highest per capita treatment costs were observed in injuries and poisoning (89,000 yuan). In 2021, the costliest treatments were for meningitis and encephalopathy (133,000 yuan); in 2022, injuries and poisoning (91,000 yuan); and in 2023, pregnancy, childbirth, and perinatal diseases (88,000 yuan) (Table 3).

## Influencing factors of medical costs

### Total medical expenditures

The results of multiple linear regression analysis indicated that the number of surgeries, length of hospital stay, interdepartmental transfer, age, urban employee medical insurance, and presence of comorbidities were all positively correlated with total hospitalization costs. Conversely, cardiovascular diseases, malignant tumors, and digestive system diseases were negatively correlated with total medical expenditures. These associations were statistically significant. The adjusted coefficient of determination R^2^ was 49.7%, indicating that the regression model is statistically significant (*P* < 0.05) (Table 4).

**Table 4:** Multiple linear regression analysis results of influencing factors of total medical expenditures

	B	95% CI of B		Sig.
		Lower	Upper	
(constant )	3.784	3.633	3.935	0.000
No. of surgical procedures	0.174	0.166	0.182	0.000
ALOS	0.006	0.005	0.006	0.000
Cardiovascular disease or not	-0.361	-0.399	-0.323	0.000
Interdepartmental transfer or not	0.131	0.093	0.170	0.000
Age	0.002	0.001	0.003	0.000
Cancer or not	-0.097	-0.144	-0.050	0.000
URRBMI or not​	0.090	0.047	0.134	0.000
Digestive system disease or not	-0.087	-0.148	-0.027	0.005
Comorbidity or not	0.147	0.015	0.279	0.030

ALOS: Average Length of Hospital Stay

URRBMI: Urban and rural residents basic medical insurance

Dependent variable: Total medical expenditures

The method of independent variable selection is stepwise

### OOP payments

The multiple linear regression analysis also revealed that the number of surgeries, length of hospital stay, interdepartmental transfer, urban–rural insurance, sex, and a primary diagnosis of cerebrovascular disease or respiratory disease, as well as marital status (spouse present), were positively associated with increased OOP payments, demonstrating statistically significant differences. In contrast, cardiovascular disease, years of hospitalizations, number of previous hospitalizations, drug allergy history, and age were negatively correlated with OOP payments. The adjusted R^2^ was 46.4%, confirming that the regression model was statistically significant (*P* < 0.05) (Table 5).

**Table 5:** Multiple linear regression analysis results of influencing factors of Out-of-pocket payments

	B	95% CI of B		Sig.
		Lower	Upper	
(constant )	96.273	56.384	136.162	0.000
No. of surgical procedures	0.168	0.160	0.177	0.000
ALOS	0.005	0.005	0.006	0.000
Cardiovascular disease or not	-0.225	-0.272	-0.178	0.000
Interdepartmental transfer or not	0.151	0.107	0.195	0.000
No. of hospitalizations at this hospital	-0.007	-0.01	-0.004	0.000
URRBMI or not​	0.263	0.155	0.371	0.000
Year	-0.046	-0.066	-0.026	0.000
Sex	0.065	0.031	0.098	0.000
Cerebrovascular disease or not	0.083	0.038	0.127	0.000
Respiratory disease or not	0.084	0.042	0.127	0.000
Drug allergy	-0.094	-0.164	-0.024	0.009
Age	-0.002	-0.003	0.000	0.007
Have partner	0.060	0.010	0.110	0.020

ALOS: Average Length of Hospital Stay

URRBMI: Urban and rural residents basic medical insurance

Dependent variable: Out-of-pocket payments

The method of independent variable selection is stepwise

## Discussion

Patients aged ≥ 65 years accounted for the majority of in-hospital deaths (73%) and represented 84.5% of total medical costs. Age at death was positively correlated with total hospitalization costs but negatively correlated with OOP payments. By the end of 2023, China’s population aged 65 and older had reached 200 million, representing 15.4% of the total population. With a decline in the total year-end population (a reduction of 2.08 million between 2022 and 2023) [4] and an increase in life expectancy (78.6 years) [11]_,_ the proportion of deaths among individuals aged 65 and above is expected to remain high in the coming years. This aging population faces multiple challenges, chronic diseases [12], infectious diseases [13, 14], disabilities [15], and mental health disorders [16]. The increasing number of EOL patients will impose continuous pressure on healthcare systems and medical expenditures. Previous studies confirmed a substantial rise in medical costs [17–19], particularly among the elderly [20]. The observed negative correlation between age and OOP payments may be attributed to broader insurance coverage and potential overutilization of hospital-based EOL care. To address these challenges, several implementable strategies are recommended: establishing health monitoring systems for the elderly, promoting long-term care insurance [21], enhancing psychological and health education services, and encouraging families and communities’ participation in caregiving. These approaches could help alleviate the financial and systemic burden on both families and the healthcare infrastructure.

The average medical cost was ¥65,000, substantially higher than the 2023 national public hospital average of ¥12,900. Although in-hospital deaths represented only 1.0% of all inpatients, they accounted for 2.6% of total hospitalization expenditures. The average OOP payments during the final hospitalization constituted 35.2% of the local per capita disposable income, highlighting a considerable financial burden on patients and their families. Western medicine (32.4%) and diagnostic testing (15.3%) accounted for the largest portions of medical expenses, differing from the findings of Yang Tongling and Zhu Yuting [22, 23], likely due to variations in study population and potential over-treatment. Despite notable progress in healthcare reform, China continues to face the dual challenges of escalating medical costs and increasing financial strain on patients [24]. Policymakers should therefore pursue context-specific reforms, strengthen the role of pharmacy in primary health care, enhance diagnostic stewardship to optimize disease identification and treatment [25–27], and promote cost-effective clinical pathways.

The presence of a spouse was positively associated with higher OOP paymentws, possibly reflecting emotional influences that encourage more aggressive EOL treatments [28, 29]. Patients at this stage often endure profound physical and psychological distress [30–32]. Similar trends have been observed internationally, such as in the United States, where life-prolonging interventions are frequently prioritized [33, 34]. As spouses play an increasingly central role in caregiving [35], complex decision-making dilemmas emerge concerning EOL care [36, 37]. To support dignified EOL experiences, community health institutions should develop standardized palliative-care programs, introduce early palliative-care triggers, and provide specialized training in EOL support. Public education initiatives should foster realistic attitudes toward death and terminal care [38]. Furthermore, expanding home-based palliative care [39] and offering psychological support for spouses can mitigate unnecessary interventions and reduce the risk of medical impoverishment, thereby contributing to a comprehensive social support framework [40].

In-hospital deaths covered by urban employee medical insurance incurred higher total medical costs compared to other insurance types, suggesting that this insurance may incentivize over-treatment. In contrast, urban-rural insurance death incurred lower self-paid costs, likely reflecting more conservative care approaches. Similar observations have been reported by Xiao Jiuqing et al. [41]. Disparities in insurance design contribute to unequal cost-sharing structures [42, 43]. As China advances toward integration of its health insurance systems, stronger oversight of insurance fund utilization, both by providers and beneficiaries, is essential. Further reforms should encourage rational service utilization and ensure sustainable cost control.

Comorbidities were significantly increased in both total costs and OOP payments. Among the deceased patients, 98.9% had secondary diagnoses, with a median of eight comorbidities. Individuals with multiple health conditions face complex care requirements, repeated diagnostic testing, polypharmacy, and higher risks of adverse drug reactions [44, 45]. Given that 74.6% of adults aged 60 years and above have comorbidities [46], effective management is imperative. China should therefore establish standardized multimorbidity management pathways [47, 48], strengthen screening and coordinated care for high-risk groups, and enhance treatment efficiency through integrated care models to control medical costs. As population aging accelerates and disease patterns shift, multidisciplinary strategies addressing medical, social, and psychological aspects will be vital for sustainable healthcare delivery.

### Limitations

This study has several limitations. First, the data were obtained from a single tertiary hospital over a three-year period, which limits the overall sample size. Additionally, the range of variables was constrained because the data were extracted solely from medical records, potentially omitting other influential factors. Second, information on patients’ family incomes could not be collected; therefore, preventing an assessment of whether medical expenditures had reached the threshold for catastrophic health spending. Future studies should include data from multiple hospitals and regions, incorporate a broader range of variables, and employ mixed-method approaches, including qualitative interviews, to provide a more comprehensive understanding of EOL healthcare utilization and costs.

## Conclusions

China’s aging population has intensified the challenges associated with EOL medical expenditure and healthcare utilization. Policymakers should implement comprehensive reforms, addressing both supply and demand aspects of healthcare alongside educational campaigns that promote a balanced understanding of life and death and EOL care. Furthermore, it is essential to strengthen patient-centered mechanisms such as long-term care insurance, home-based palliative services, and standardized comorbidity management systems. Concurrently, reforms in hospital operations and medical insurance should emphasize transparency and strengthen supervision and equitable access. Greater involvement of communities and social organizations in providing care and emotional support for patients at EOL. Collectively, these efforts substantially improve the quality of life of such patients, promote more efficient use of healthcare services, and contribute to the sustainable containment of medical cost escalation.

## Data Availability

The data that support the findings of this study are available from the corresponding author，upon reasonable request.
